# Advanced age-related macular degeneration and risk factors in eyes with pachydrusen

**DOI:** 10.1038/s41598-024-56404-8

**Published:** 2024-03-13

**Authors:** Seung Wan Nam, Hoon Noh, Je Moon Yoon, Don-Il Ham

**Affiliations:** 1 Good Morning Light Eye Clinic, Ulsan, Korea; 2 Department of Ophthalmology, HanGil Eye Hospital, Incheon, Korea; 3 Department of Ophthalmology, College of Medicine, Catholic Kwandong University, Incheon, Korea; 4 Department of Ophthalmology, Samsung Medical Center, Sungkyunkwan University School of Medicine, Seoul, Korea

**Keywords:** Diseases, Medical research, Risk factors

## Abstract

The risk of progression to advanced age-related macular degeneration (AMD) varies depending on the type of drusen. This retrospective longitudinal study included 248 eyes of 156 patients with pachydrusen without advanced AMD at baseline. Macular neovascularization (MNV) and geographic atrophy (GA) were evaluated. Risk factors for progression to advanced AMD were determined using multivariate Cox regression analysis. The mean age at baseline was 65.4 ± 9.1 years, and the mean follow-up duration was 6.40 ± 3.58 years. The mean total number of pachydrusen and macular pachydrusen were 4.10 ± 2.85 and 2.27 ± 1.81 per eye, respectively. Pachydrusen was accompanied by other types of drusen in 4.8% (12 eyes) of eyes at baseline. During follow-up, MNVs occurred in 2.8% (seven eyes), including polypoidal choroidal vasculopathy (PCV six eyes); however, no GA occurred. Regarding risk factors for progression to neovascular AMD, age (*p* = 0.023) and macular pigmentary changes (*p* = 0.014) were significantly associated with MNV development. The cumulative incidence of MNV was significantly higher in the group with macular pigmentary changes (17.39% vs. 0.57% at 10 years; *p* = 0.0005). The number of macular pachydrusen and the presence of MNV in the fellow eye did not show a statistically significant relationship with MNV development. Age and macular pigmentary changes are risk factors for MNV development in the eyes with pachydrusen. Eyes with pachydrusen appear to have a risk profile for advanced AMD that is different from that of AMD eyes with drusen or drusenoid deposits other than pachydrusen.

## Introduction

Age-related macular degeneration (AMD) is the leading cause of blindness in developed countries^1^. Early stage AMD can progress to advanced AMD, including geographic atrophy (GA) and macular neovascularization (MNV), which are associated with visual impairment^2^. Drusen and macular pigmentary changes are known risk factors for advanced AMD. Recently, reticular pseudodrusen (RPD) has also been considered a risk factor for this condition^3^. In addition, it has been reported that there is a difference in the occurrence of MNV type according to the type of drusen present, such as a more frequent occurrence of type 3 MNV in eyes with RPD^4–6^.

Pachydrusen is a recently identified type of drusen, which have well-defined margins with an irregular outer contour and which occur in isolation or in groups of a few at the posterior pole^7^. The prevalence of pachydrusen was reported as 7.7% in a Japanese population aged over 40 years^8^. In hospital-based studies, the prevalence of pachydrusen was 25.5% in Singaporean people, 11.7% in White people, and 8.4% in Indian people with nonexudative AMD^9^. While advanced AMD occurred in eyes with pachydrusen, GA was rarely observed in these eyes^8^. The 5-year incidence of MNVs in non-neovascular AMD eyes with pachydrusen was 0–17.0%^8,10,11^. Moreover, pachydrusen was frequently present in eyes with polypoidal choroidal vasculopathy (PCV), and PCV more frequently occurred in eyes with pachydrusen than in eyes with other types of drusen^10,11^. However, many aspects of pachydrusen, including the long-term prognosis and risk factors for progression to advanced AMD remain unclear, due to a paucity of a longitudinal study of pachydrusen^10–12^.

This study aimed to investigate the occurrence of advanced AMD and risk factors for progression to advanced AMD in eyes with pachydrusen.

## Methods

The medical records of consecutive patients aged > 50 years who were diagnosed with intermediate AMD at the retinal clinic of the Samsung Medical Center, Seoul, Korea, between January 2006 and June 2021, were retrospectively reviewed. The eyes of patients who had been followed up for more than 1 year with multimodal imaging were assessed. Eyes with pachydrusen without GA or MNV were included. The exclusion criteria were advanced AMD present at the first visit (i.e., presence of GA or MNV), uveitis, severe diabetic retinopathy, severe hypertensive retinopathy, severe epiretinal membrane, retinal detachment, glaucoma, refractive error exceeding 6 diopters (D), history of retinal laser photocoagulation, severe ocular media opacity, and insufficient ocular examinations.

This study was reviewed and approved by the Institutional Review Board of the Samsung Medical Center. This study was conducted in accordance with the principles of the Declaration of Helsinki. The requirement for informed consent was waived because of the retrospective nature of the study and because the analysis used anonymous clinical data.

Basic demographic information including age, sex, and comorbidities was collected at the first visit. All patients underwent a comprehensive ophthalmologic evaluation, including slit-lamp examination, best-corrected visual acuity assessment, intraocular pressure measurements, and fundus examination with pupil dilation. Color fundus photography (CFP), fundus autofluorescence (FAF), infrared imaging, and spectral domain optical coherence tomography (SD-OCT) were performed in all patients. CFP was performed using a fundus camera (TRC 50 IX or DX; Topcon, Tokyo, Japan) with a 50′ field of view. FAF, IR, and SD-OCT images were acquired using a Spectralis HRA + OCT (Heidelberg Engineering, Heidelberg, Germany). The protocol for SD-OCT (Spectralis HRA + OCT, Heidelberg Engineering, Heidelberg, Germany) consisted of two B-scans centered on the fovea (horizontal and vertical, 12.0 mm, ART 100) and raster scans (30′ × 25′, 9.0 mm, centered on the fovea, 31 horizontal B-scans, ART 25) using enhanced-depth imaging (EDI) protocols^13^. Automatic real-time (ART) mode using the eye-tracker system was activated. Fluorescein angiography (FA) and indocyanine green angiography (ICGA) were performed (Spectralis HRA + OCT or Optos 200Tx, Optos PLC, Dunfermline, UK) if neovascular changes were observed on OCT images. The subfoveal choroidal thickness (SFCT) was measured manually at the foveal center using a horizontal B-scan image from EDI-OCT. The macula was defined using a radius of 3000 µm within the outermost ring of the Early Treatment Diabetic Retinopathy Study (ETDRS) grid centered at the fovea^14^.

All images were assessed by two investigators (S.W.N. and H.N.). Inter- and intragrader agreement on each fundus feature was regularly assessed, and consensus training was initiated when κ values were below 0.6. A senior interpreter adjudicated all uncertain interpreter (D-I.H.).

### Definition of the drusen subtypes

Pachydrusen were defined as large drusen (> 125 μm) with an irregular border on CFP images and the deposit below the layer of the retinal pigment epithelium (RPE) in EDI-OCT^7^. The number of pachydrusen was counted using CFP, and clusters of pachydrusen were counted as one pachydrusen. Pachydrusen in the macula were also counted according to the AMD grading method reported by the AREDS study group^15^.

Soft drusen were defined as round or ovoid-shaped drusen with a poorly defined border that might be tightly packed and even confluent on CFP images, with deposits below the RPE layer on EDI-OCT images^7^.

RPD were defined as follows: (1) multiple yellowish-white lesions with a reticular network in CFP, (2) an interlacing network in red-free imaging, (3) hyporeflectant lesions with mild background hyperreflectance in near-infrared imaging, (4) hypofluorescent lesions against a background of mild hyperfluorescence in FAF imaging, (5) ≥ 5 hyperreflective subretinal deposits above the RPE on more than one B-scan image in OCT, and (6) hypofluorescent lesions in the mid- or late-phase of ICGA. RPD were considered present if they were identified in at least three imaging methods, including OCT^16^.

Cuticular drusen were defined as multiple, yellow, or pale, small, round lesions observed in the CFP that showed a symmetrical distribution pattern between the bilateral eyes. There had to be at least 50 scattered, uniformly sized, small (25–75 μm) hyperfluorescent drusen with a typical “stars-in-the-sky” appearance on FA images in each eye^17,18^. The lesion had to be located beneath the RPE, with RPE elevation noted on OCT images^17,19^.

### Definition of macular pigmentary change and macular RPE abnormalities

Macular pigmentary changes (none, hyperpigmentation, or hypopigmentation) were identified on the CFP images. Macular RPE abnormalities [none, RPE irregularity, and pigment epithelial detachment (PED)] were identified on the OCT images. RPE irregularity was defined as an irregular elevation of the RPE above the Bruch’s membrane. PED was defined as a dome-shaped elevation of the RPE above Bruch’s membrane.

### Definition of advanced AMD

The definition and classification of neovascular AMD followed the criteria proposed by the Consensus on Neovascular Age-related macular degeneration Nomenclature study group^20^. MNVs were subdivided into four types: MNV types 1, 2, and 3, and PCV. MNV subtypes were comprehensively diagnosed based on CFP, FA, ICGA, and OCT findings. PCV diagnosis was made using the EVEREST study criteria, which is focusing on the presence of focal hyperfluorescent lesions (aneurysms/polyps) appearing on ICGA before minute 6, associated with a branching vascular network/type 1 MNV^21^.

GA was defined as a sharply demarcated hypopigmented area with large visible choroidal vessels in CFP and hypoautofluorescence in FAF, with a diameter of at least 175 μm^22^.

### Statistical analyses

For data analyses, we employed the independent sample *t*-test for continuous variables and the chi-squared test and Fisher's exact test for nominal variables to identify the risk factors for progression to neovascular AMD and compare the clinical characteristics based on macular pigmentary changes. Cumulative incidence curves and log-rank tests were used to analyze the development of MNV according to macular pigmentary changes. The Cox proportional hazard model was used for the univariate regression analysis of the clinical factors associated with neovascular changes. A multivariate Cox regression analysis was performed for significant factors that had a *p*-value < 0.15 in the univariate analysis. Statistical significance was defined as a *p*-value < 0.05. All the statistical analyses were performed using SAS version 9.4 (SAS Institute, Cary, NC, USA).

### Ethics statement

This study was reviewed and approved by the Institutional Review Board of Samsung Medical Center (IRB No: 2022-01-160). This study was conducted in accordance with the principles of the Declaration of Helsinki. The requirement for informed consent was waived because of the retrospective nature of the study and because the analysis used anonymous clinical data.

## Results

Of the 4204 eyes of 2187 patients aged > 50 years who were referred to the retinal clinic in our hospital, 967 eyes of 494 patients were identified with drusen or drusenoid deposits via multimodal imaging. Among these, 288 eyes of 156 patients had pachydrusen (288/967 eyes; 29.8%). After 40 eyes were excluded because of accompanying MNV (40 eyes), 248 eyes from 156 patients were finally enrolled in this study. At baseline, the mean age was 65.4 ± 9.1 years (range 50.0–93.8 years). A total of 130 eyes belonged to 84 male patients and 118 eyes belonged to 72 female patients. The mean follow-up duration was 6.40 ± 3.58 years (range 1.00–13.21 years). More than half of the eyes were followed up for 5 years or longer: 82.7% (205/248 eyes), 62.9% (156/248 eyes), 42.7% (106/248 eyes), and 17.7% (44/248 eyes) had follow-ups of 3, 5, 7, and 10 years or longer, respectively.

Total 1016 pachydrusen were found in 248 eyes, and the mean number of pachydrusen per eye was 4.10 ± 2.85 (range 1–14) per eye. A total of 564 pachydrusen (55.5%) were in the macula and 452 (44.5%) were located in the extramacula. Regarding the number of eyes according to the location of pachydrusen, 104 eyes (41.9%) had pachydrusen only in the macula, 90 eyes (36.3%) had pachydrusen only in the extramacula, and 54 eyes (21.8%) had pachydrusen in both the macula and extramacula. During the follow-up period, the mean number of macular pachydrusen increased to 2.97 ± 2.49 per eye at a rate of 0.15 ± 0.74 per eye/year. Only one eye showed pachydrusen regression, and the number of pachydrusen decreased from 1 to 0.

Other types of drusen coexisted in 4.8% of the eyes (12 eyes of seven patients). Among these eyes, nine eyes from five patients had accompanying soft drusen, and three eyes from two patients had accompanying cuticular drusen. Macular pigmentary changes were concurrently observed in 27.4% of eyes (68/248). Regarding macular RPE abnormalities, 51 eyes showed RPE irregularities and 17 eyes showed PED (Table 1).

**Table 1:** Baseline clinical characteristics of eyes with pachydrusen without geographic atrophy or macular neovascularization.

	Pachydrusen eyes (n = 248)
Age (years)	65.4 ± 9.1
Male (%)	130 (52.4%)
Refractive error (D) (spherical equivalent)	0.15 ± 1.51
Best-corrected visual acuity (logMAR)	0.08 ± 0.17
Intraocular pressure (mmHg)	15.3 ± 3.1
Lens status (phakia/pseudophakia)	227 (91.5%)/21 (8.5%)
Subfoveal choroidal thickness (μm)	294.4 ± 94.3
Number of macular pachydrusen per eye	2.27 ± 1.81
Presence of other drusen (%)	12 (4.8%)
Soft drusen	9
Reticular pseudodrusen	0
Cuticular drusen	3
Macular pigmentary changes (%)	68 (27.4%)
Macular RPE changes (%)	68 (27.4%)
RPE irregularity	51
PED	17
Fellow eye with MNV/GA (%)	40 (16.1%)/0 (0.0%)

Data are total no. (%) or mean ± standard deviation, unless otherwise indicated.

*GA* Geographic atrophy, *MNV* macular neovascularization, *D* Diopters, *logMAR* logarithm of the minimum angle of resolution, *RPE* retinal pigment epithelium, *PED* pigment epithelial detachment.

**Figure 1:**
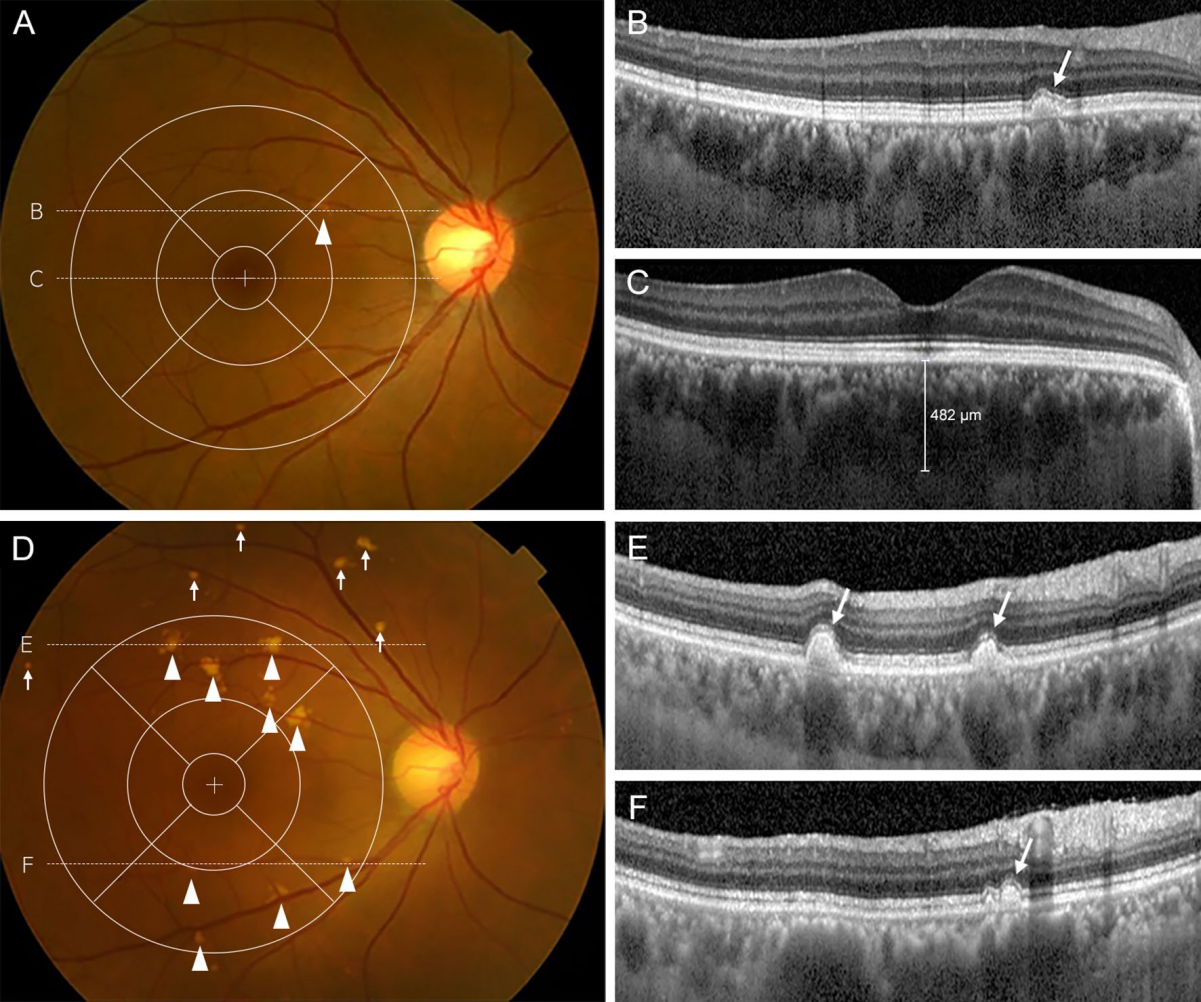
Pachydrusen eye without macular pigmentary changes. Color fundus photograph of a 51-year-old woman showing pachydrusen located in the macula (arrowhead) (**A**). Other types of drusen, macular pigmentary changes, macular neovascularization (MNV), and geographic atrophy (GA) are absent. A spectral-domain optical coherence tomography (SD-OCT) scan at the position of the pachydrusen (dashed white line **B**) demonstrates a subretinal pigment epithelial (subRPE) deposit (arrow) (**B**). SD-OCT scan of the fovea (dashed white line **C**) demonstrating subfoveal choroidal thickening (**C**). After 12 years, color fundus photography showed an increased number of pachydrusen at the macula (arrowheads) and extramacular area (arrows) and no MNV or GA (**D**). SD-OCT scans at the pachydrusen (dashed white lines **E** and **F**) showing subRPE deposits (arrows) (**E**, **F**).

**Figure 2:**
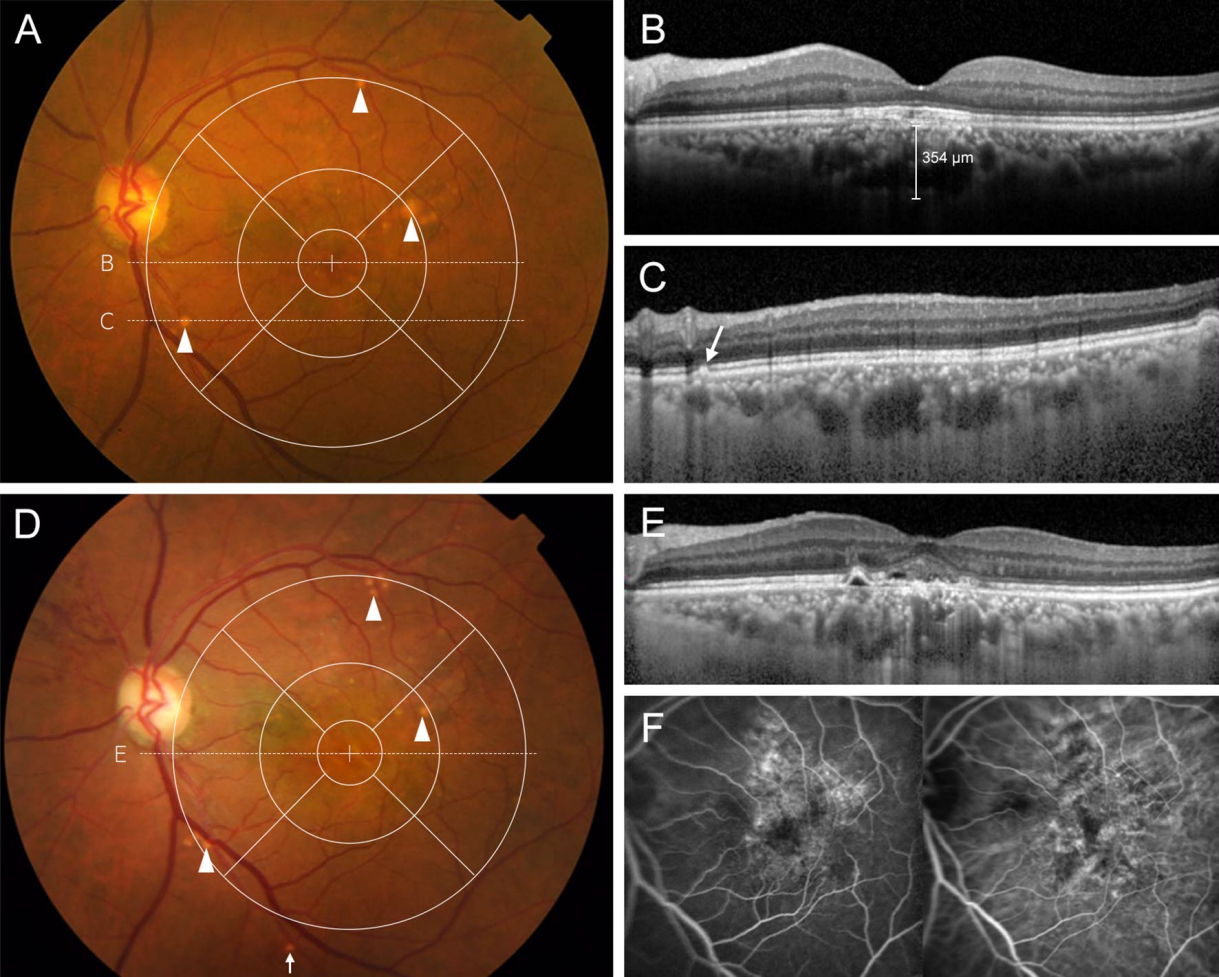
Pachydrusen eye with macular pigmentary changes. Color fundus photograph of a 73-year-old man showing three pachydrusen and macular pigmentary changes in the macula (arrowhead) (**A**). Other types of drusen, macular neovascularization (MNV), and geographic atrophy (GA) are absent. Spectral-domain optical coherence tomography (SD-OCT) scan at the fovea (dashed white line **B**) demonstrates subfoveal choroidal thickening (**B**). An SD-OCT scan at the position of the pachydrusen (dashed white line **C**) demonstrating subretinal pigment epithelial deposits (arrow) (**C**). Eight years after the first visit, color fundus photography reveals three pachydrusen in the macula (arrowheads) and one pachydrusen in the extramacular area (arrow) (**D**). SD-OCT reveals the presence of subretinal fluid and tissue, indicating the development of MNV (**E**). Fluorescein angiography shows stippled hyperfluorescence characteristic of occult MNV (**F**), and indocyanine green angiography shows hyperfluorescent spots with hypofluorescent lesions (**G**).

MNV occurred in 2.8% (7/248) of cases, but no GA occurred. The MNV types were PCV in six eyes and type 1 MNV in one eye. MNV types 2 and 3 were not observed during follow-up. Representative cases are shown in Figs. 1 and 2.

### Risk factors for progression to neovascular AMD

In the univariate Cox regression analyses of risk factors for progression to neovascular AMD, sex, other concurrent drusen, number of macular pachydrusen, and presence of neovascular AMD in the fellow eye at baseline were not associated with the development of MNV (*p* > 0.15). However, age, SFCT, and macular pigmentary changes were significantly associated with MNV development (*p* < 0.15). Therefore, we performed multivariate analyses with age, SFCT, and macular pigmentary changes. In multivariate Cox regression analysis, older age (*p* = 0.023) and the presence of macular pigmentary changes (*p* = 0.014) significantly increased the risk of progression to neovascular AMD (Table 2).

**Table 2:** Factors associated with progression to neovascular age-related macular degeneration.

Variables	Hazard ratio	95% CI	*p*-value
Univariate Cox regression analysis			
Age	1.161	1.045–1.289	0.005
Sex (Male = 1, Female = 2)	0.655	0.125–3.438	0.617
Other drusen (None = 1, Present = 2)	3.715	0.433–31.881	0.232
Subfoveal choroidal thickness	0.993	0.984–1.002	0.118
Macular pigmentary changes	15.938	1.918–132.428	0.010
Number of macular pachydrusen	1.114	0.701–1.770	0.647
MNV in the fellow eye (Without MNV = 1, With MNV = 2)	0.894	0.107–7.444	0.918
Multivariate Cox regression analysis			
Age	1.143	1.019–1.282	0.023
Subfoveal choroidal thickness	0.996	0.988–1.004	0.303
Macular pigmentary changes	14.734	1.737–124.954	0.014

Data are total no. (%) or mean ± standard deviation, unless otherwise indicated.

*AMD* Age-related macular degeneration, *MNV* Macular neovascularization, *CI* confidence interval.

**Figure 3:**
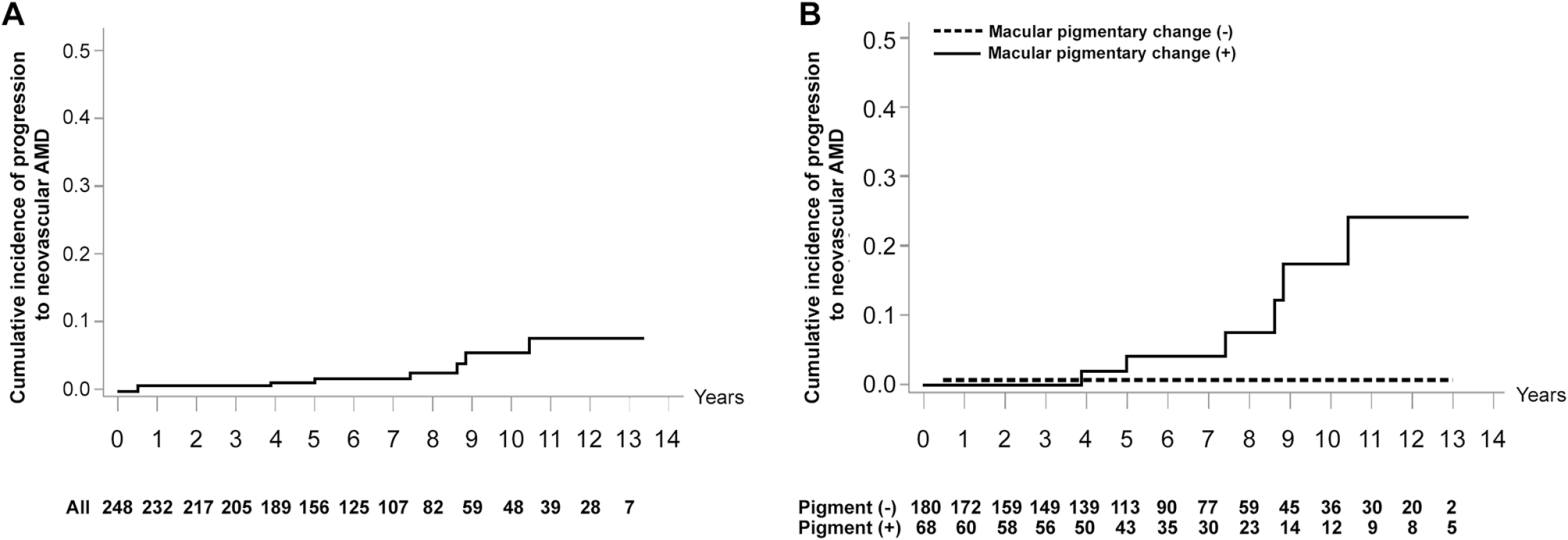
Cumulative incidence curves and log-rank test of macular neovascularization (MNV) occurrence in eyes with pachydrusen. (**A**) Cumulative incidence curve for the time taken to develop MNV in all participants. The cumulative incidence of progression to neovascular age-related macular degeneration (AMD) in eyes with pachydrusen is 1.57% at 5 years and 5.46% at 10 years of age. (**B**) Cumulative incidence curves for the time to develop MNV, stratified by the presence of macular pigmentary changes at baseline. The cumulative incidence of neovascular AMD in patients with macular pigmentary changes was 4.19% at 5 years and 17.39% at 10 years of age. The cumulative incidence of neovascular AMD in patients without macular pigmentary changes was 0.57% and 0.57% at 5 and 10 years, respectively. The log-rank test revealed a significant difference between the curves (*p* = 0.0005). Hazard ratios, 95% confidence intervals, and *p*-values are displayed for individual covariates.

The cumulative incidence of progression to neovascular AMD in eyes with pachydrusen was 1.57% over 5-year period and 5.46% over a 10-year period. In terms of age, the frequency of MNV development in patients older than 67 years was significantly greater than in those aged 67 years or younger (5.5% vs. 0.7%, *p* = 0.047), although they had a short period of follow-up (5.36 ± 3.42 years vs. 7.22 ± 3.51 years, *p* < 0.001) (Supplemental Table 1). In the macular pigmentary changes, the cumulative incidence of neovascular AMD in the group with macular pigmentary changes was 4.19% over a 5-year period and 17.39% over a 10-year period. The cumulative incidence of neovascular AMD in the group without macular pigmentary changes was 0.57% over both 5 years and 10 years. The log-rank test revealed significant differences between the curves (*p* = 0.0005) (Fig. 3). Comparative analyses of eyes with pachydrusen with and without macular pigmentation changes were performed. Follow-up duration was not significantly different between the two groups (6.35 ± 3.77 years vs. 6.41 ± 3.52 years, *p* = 0.897). Age, number of macular pachydrusen at baseline and last follow-up, increase in the number of macular pachydrusen per year, and SFCT were not significantly different between the two groups (*p* > 0.05). However, MNV developed in 8.8% of the eyes with macular pigmentary changes and 0.5% of the eyes without macular pigmentary changes (*p* = 0.002). The proportion of men was significantly higher among those with macular pigmentary changes (67.6% vs. 46.7%; *p* = 0.003) (Supplemental Table 2).

## Discussion

This study investigated the development of advanced AMD and its risk factors in eyes with pachydrusen. Previous studies have reported the longitudinal changes in pachydrusen. The number of eyes with pachydrusen and the mean follow-up duration in those studies were 29–140 eyes and 3.71–5 years^8,10–12^. This study included a larger number of study eyes and a longer mean follow-up duration (248 eyes over 6.40 years) than did the previous studies.

The incidence of MNV was low in this study. It was 2.8% over the mean duration of 6.4 years, and the cumulative incidence of progression to neovascular AMD over a 5-year period was 1.57%. The results of several previous studies support the low incidence of MNV observed in the present study. A population-based study in Japan reported that no eyes with pachydrusen progressed to late AMD within the 5-year follow-up period^8^. A fellow eye study of unilateral exudative AMD reported no occurrence of exudative AMD in 40 pachydrusen eyes over the mean duration of 47.1 months^23^. Another fellow eye study of unilateral exudative AMD also reported 7.1% of 5-year incidence, which was far less than that of soft drusen plus pseudodrusen eyes (76.4%) and soft drusen eyes (46.2%)^11^. However, a longitudinal study of pachydrusen reported 17% of the 5-year incidence of MNV^10^. A small study also reported 24.1% over the mean duration of 4.2 years^12^. Possible explanations for these differences are as follows. One is that the mean age of eyes with pachydrusen in this study was younger (65.4 years) than that in previous studies (66.3 years ≤) and age is an independent risk factor for progression to neovascular AMD^10,24^. Another is that there might be some inaccurate diagnosis of pachydrusen, which induced the increase of incidence rate. The incidence of MNV in pachydrusen eyes is lower than that in eyes with soft drusen or reticular pseudodrusen^8,11,23^. Thus, eyes with other drusen misdiagnosed as pachydrusen could increase the incidence rate of MNV. Another explanation is that there may be some unknown risk factors related to the incidence of MNV in eyes with pachydrusen. In AMD eyes with large drusen, the 5-year incidence of progression to advanced AMD ranges widely from 3.9% to 47.3%, depending on the presence of risk factors in both eyes^15^. A similar situation might occur in pachydrusen eyes. We suppose that the incidence of MNV in pachydrusen eyes is not higher and probably lower than that in soft drusen eyes. Three previous studies and the present study found a low incidence of MNV in pachydrusen eyes, and two previous studies reporting a high incidence of MNV found no significant difference in the incidence of MNV between pachydrusen eyes and soft drusen eyes^8,10–12,23^.

In this study, PCV was the predominant type of MNV (6 eyes, 85.7%), which is consistent with that of previous studies that reported that PCV was the predominant type of MNV in eyes with pachydrusen^10,11^. In previous longitudinal studies of eyes with pachydrusen, PCV accounted for 45.0–77.8% of MNV, and no type 3 MNV occurred^10,11^.

Drusen and macular pigmentary changes are two important diagnostic markers of AMD and are risk factors for the development of advanced AMD^15,22,24^. In AMD, the size, number, area, and volume of drusen are related to the development of MNV^25,26^. Other risk factors are age and the presence of MNV in the fellow eye^27^. In this study, age and macular pigmentary changes were risk factors for the progression to neovascular AMD in eyes with pachydrusen. However, neither the number of macular pachydrusen nor the presence of MNV in the fellow eye at baseline was associated with the development of MNV.

In the analysis of MNV development according to the age at baseline, those aged older than 67 years showed a higher frequency of MNV development than did those aged 67 years or younger (*p* = 0.047), although they had a short period of follow-up. However, considering the small number of MNV cases, further large-scale longitudinal studies are warranted.

To analyze MNV development according to the macular pigmentary changes at baseline, we analyzed the cumulative incidence curves of MNVs. The cumulative incidence curves differed significantly based on the presence of macular pigmentary changes. In eyes with pachydrusen, the 10-year cumulative incidence of MNV was significantly higher when macular pigmentary changes were present than when they were absent (17.39% vs. 0.57%). A comparison of groups with and without macular pigmentary changes showed similar results, although the probability of MNV occurrence between the two groups was similar at baseline.

In AMD eyes, the 5-year incidence of GA was from 0.6%–15% depending on the risk scores^28^. Age, smoking, hypertension, AREDS score, and night vision score were reported as risk factors for GA in AMD^28^. However, GA did not occur in eyes with pachydrusen during the follow-up period in this study. This result is consistent with that of a previous pachydrusen study that reported no development of GA^8,28^. Another study also reported that GA was rarely observed after focal disruption of the ellipsoid zone at pachydrusen sites^29^. Thus, pachydrusen appears to be unrelated to the development of GA.

This longitudinal study indicated that eyes with pachydrusen had a risk profile for progression to advanced AMD that differed from that of AMD eyes without pachydrusen. A low incidence of MNV, no association with progression to GA, and no significant risk of neovascular AMD associated with the macular drusen number or MNV in the fellow eye were not typical features of AMD. In a previous fellow eye study of unilateral neovascula AMD, pachydrusen was not shown to be a significant risk factor compared to the no drusen group^11^. Another fellow eye study reported risk allele frequency of ARMS2 A69S was significantly lower in pachydrusen eyes than in soft drusen eyes and in pseudodrusen eyes^23^. In addition, most of pachydrusen eyes have no other drusen, and most of the pachydrusen were located outside the macula area^8,9^.

Advanced AMD-risk prediction is useful for managing patients with non-exudative AMD, and many methods can be used in clinical practice, including a simplified severity scale and an advanced AMD risk calculator^15,27^. It is noteworthy that current advanced AMD risk prediction methods are based on the assumption that every kind of drusen has the same risk of progression to advanced AMD. However, a recent post-hoc analysis of AREDS and AREDS2 raised questions about the same risk for various drusen, emphasizing the necessity of  inclusion of RPD status in AMD classification systems, risk calculators, and clinical trials^3^.

For a similar reason, if pachydrusen eyes have a risk profile for progression to advanced AMD that is different from that of AMD eyes with drusen other than pachydrusen, the current advanced AMD risk prediction methods may not work in eyes with pachydrusen^15^. Moreover, in any clinical AMD study on the risk of advanced AMD, the results may be affected by the proportion of study participants with pachydrusen. Pachydrusen is more prevalent than soft drusen and RPD in Asian patients, and the prevalence of pachydrusen is higher in Asian patients than in White patients^8,9,11^. Thus, the proportion of pachydrusen should be considered in clinical AMD studies, especially in Asian patients. We believe that the current AMD classification should be updated to distinguish drusen types if pachydrusen has a different risk profile from that of other drusen. Further studies with larger sample sizes should be performed to confirm the results of this study.

This study has some limitations. This was a single-hospital retrospective study. No comparisons were made between the study and control eyes. Patients aged < 50 years were excluded. A limited number of patients were followed-up for various observation periods. The drusen size and volume were not investigated as risk factors. In counting the number of pachydrusen, only pachydrusen in the inside of the outermost ring of the ETDRS grid were counted to evaluate whether the pachydrusen is a risk factor for the progression to MNV. We aimed to determine whether pachydrusen could be included in the current advanced AMD risk calculator. Clusters of pachydrusen were counted as one pachydrusen; thus, the number of pachydrusen was not linearly proportional to the area of the pachydrusen. The presence of other drusen in eyes with pachydrusen was not associated with the occurrence of MNV (*p* > 0.05). However, the statistical power of the study was low, because only a small number of eyes had other types of drusen. This study was performed in a tertiary referral hospital; therefore, a selection bias may have been present.

In conclusion, the occurrence of GA is probably not associated with the presence of pachydrusen, although MNV, predominantly PCV, occurs in eyes with pachydrusen. Age and macular pigmentary changes, but not the number of pachydrusen or the presence of MNV in the fellow eye, are risk factors for progression to neovascular AMD in eyes with pachydrusen. Eyes with pachydrusen appear to have a risk profile for the development of advanced AMD that differs from that of AMD eyes with drusen or drusenoid deposits other than pachydrusen.

### Supplementary Information

Supplementary Table 1.

Supplementary Table 2.

## Data Availability

The datasets generated and/or analyzed in the current study are available from the corresponding author upon reasonable request.

## Abbreviations

AMD: Age-related macular degeneration
ART: Automatic real-time
CFP: Color fundus photography
EDI: Enhanced-depth imaging
FA: Fluorescein angiography
FAF: Fundus autofluorescence
GA: Geographic atrophy
MNV: Macular neovascularization
PCV: Polypoidal choroidal vasculopathy
SD-OCT: Spectral domain optical coherence tomography
