# Supplementary material for: Advanced age-related macular degeneration and risk factors in eyes with pachydrusen
Source: Sci Rep. 2024 Mar 13;14:6132. doi: 10.1038/s41598-024-56404-8 (PMC10937650; doi:10.1038/s41598-024-56404-8)
Supplement: Supplementary file 1 — Supplementary Table 1. [file 41598_2024_56404_MOESM1_ESM.doc]

**Supplemental Table 1. Age-related variations of clinical characteristics in pachydrusen eyes**

|  | Age 67 years or younger  (n = 138) | Age older than 67 years  (n = 110) | p-value |
| --- | --- | --- | --- |
| Follow-up duration (years) | 7.22 ± 3.51 | 5.36 ± 3.42 | < 0.001 |
| MNV developed during the follow-up period | 1 (0.7%) | 6 (5.5%) | 0.047 |
| GA developed during the follow-up period | 0 (0.0%) | 0 (0.0%) | > 0.999 |
| Male (%) | 66 (47.8%) | 64 (58.2%) | 0.105 |
| Macular pigmentary changes | 39 (28.3%) | 29 (26.4%) | 0.739 |
| Number of macular pachydrusen per eye | 1.83 ± 1.13 | 2.83 ± 2.30 | < 0.001 |
| Subfoveal choroidal thickness (μm) | 303.3 ± 91.1 | 283.2 ± 97.3 | 0.099 |
| Presence of other drusen | 1 (0.7%) | 11 (10.0%) | 0.001 |
| Soft drusen | 0 | 9 |  |
| Reticular pseudodrusen | 0 | 0 |  |
| Cuticular drusen | 1 | 2 |  |
| Fellow eye with MNV | 17 (12.3%) | 23 (20.9%) | 0.068 |
| Fellow eye with GA | 0 (0.0%) | 0 (0.0%) | > 0.999 |
| Data are total no. (%) or mean ± standard deviation, unless otherwise indicated.  MNV = Macular neovascularization  GA = Geographic atrophy | | | |
