# Supplementary material for: Advanced age-related macular degeneration and risk factors in eyes with pachydrusen
Source: Sci Rep. 2024 Mar 13;14:6132. doi: 10.1038/s41598-024-56404-8 (PMC10937650; doi:10.1038/s41598-024-56404-8)
Supplement: Supplementary file 2 — Supplementary Table 2. [file 41598_2024_56404_MOESM2_ESM.doc]

**Supplemental Table 2.** Comparative analyses of pachydrusen eyes with and without macular pigmentary changes

|  | Macular pigmentary changes (+)  (n = 68) | Macular pigmentary changes (-)  (n = 180) | p-value |
| --- | --- | --- | --- |
| Follow-up duration (years) | 6.35 ± 3.77 | 6.41 ± 3.52 | 0.897 |
| MNV developed during the follow-up period | 6 (8.8%) | 1 (0.5%) | 0.002 |
| GA developed during the follow-up period | 0 (0.0%) | 0 (0.0%) | > 0.999 |
| Age (years) | 65.0 ± 9.0 | 65.5 ± 9.1 | 0.728 |
| Male (%) | 46 (67.6%) | 84 (46.7%) | 0.003 |
| Number of macular pachydrusen per eye at first examination | 2.25 ± 1.54 | 2.28 ± 1.91 | 0.898 |
| Number of macular pachydrusen per eye at last examination | 2.68 ± 1.91 | 3.08 ± 2.67 | 0.257 |
| Increase rate of macular pachydrusen number per year | 0.05 ± 0.14 | 0.19 ± 0.87 | 0.201 |
| Subfoveal choroidal thickness (μm) | 307.0 ± 109.9 | 289.6 ± 87.5 | 0.243 |
| Presence of other drusen | 6 (8.8%) | 6 (3.3%) | 0.072 |
| Soft drusen | 5 | 4 |  |
| Reticular pseudodrusen | 0 | 0 |  |
| Cuticular drusen | 1 | 2 |  |
| Fellow eye with MNV | 13 (19.1%) | 27 (15.0%) | 0.432 |
| Fellow eye with GA | 0 (0.0%) | 0 (0.0%) | > 0.999 |
| Data are total no. (%) or mean ± standard deviation, unless otherwise indicated.  MNV = Macular neovascularization  GA = Geographic atrophy | | | |
